# HPV vaccination in HIV+ adolescents and young adults induces strong HPV-specific immune responses

**DOI:** 10.1186/1471-2334-14-S2-P93

**Published:** 2014-05-23

**Authors:** V Rainone, D Trabattoni, F Penagini, V Fabiano, F Calascibetta, V Giacomet, A Vigano, M Clerici, GV Zuccotti

**Affiliations:** 1 University of Milan, Chair of Immunology, Milan, Italy

## Background

HPV-associated ano-genital infections represent the most common sexually-transmitted disease in the general population. The incidence of HPV-associated cancers has been increasing in HIV-infected patients. HPV vaccination may be an approach to reduce the risk of HPV-associated cancers in HIV-infected patients and a combined strategy of screening and vaccination may guarantee an adequate prevention of HPV-associated lesions. Immunogenicity of HPV vaccines in HIV-infected patients is still not adequately evaluated. We analyzed immunogenicity of a quadrivalent HPV vaccine in HIV-infected patients without molecular evidence of vaccine-type HPV infection focusing on HPV-specific cell mediated immunity (CMI).

## Methodology

31 ARV-treated HIV-infected adolescents (age range 28-14 years, with undetectable viremia and effective CD4 recovery) and 25 sex- and age-matched HIV-seronegative healthy controls were enrolled in the study. HPV-16/18/6/11 VLP vaccine (Gardasil®) was administered 3 times (baseline, 2 and 6 months). Immune activation (CD4/CD25/HLADRII, CD8/CD25/HLADRII), T-cell patterns and HPV-specific immune responses (CD4/IFN-γ/IL-2, CD8/IFN-γ/TNF-α, CD8/Perforin/GranzymeB) were evaluated.

## Results

HIV-infected individuals showed: 1) no changes in CD4 counts, percentage of CD4 cells and HIV viral load; 2) a significant increase in naїve CD8 T-cells, activated CD8 T-cells and in central memory CD4 and CD8 T-cells; 3) a significant reduction in terminally differentiated CD8 T-cells; 4) a significant increase in unstimulated and in HPV-specific IL2+/CD4+, IFN-γ+/CD4+, IFN-γ+/CD8+ and TNF-α+/CD8+ T-cells; and 5) a significant increase in HPV-specific Perforin- and Granzyme B-secreting CD8 T-cells. Results obtained in HIV-infected patients were comparable to those seen in HC.

## Conclusions

HPV-16/18/6/11 VLP vaccine induces strong HPV-specific cell-mediated immunity in ARV-treated HIV-infected individuals that are comparable to those observed in HIV-seronegative controls. HPV-specific CMI is likely an important component of the protective effect of this vaccine, data herein indicating that this arm of immunity is not impaired in ARV-treated HIV infected individuals.

